# Metabolic pattern changes of macrophages during cancer development and progression

**DOI:** 10.1186/s12967-026-08210-1

**Published:** 2026-05-12

**Authors:** Guanfu Liu, Binxue Wang, Shaojie Wang, Shi Li, Hua Han, Tai An

**Affiliations:** 1 Institute of Future Agriculture, Northwest A&F University, Yangling, 712100 China; 2 State Key Laboratory of Holistic Integrative Management of Gastrointestinal Cancers, Tangdu Innovative Institute of Science and Technology, Tangdu Hospital, Fourth Military Medical University, Xi’an, 710038 China

**Keywords:** Tumor-associated macrophages, Metabolic reprogramming, Tumor microenvironment, Immunometabolism, Metabolic intermediates, Cancer immunotherapy

## Abstract

**Background:**

Tumor-associated macrophages (TAMs) are critical components of the immune cell population within the tumor microenvironment (TME), where they play dynamic and multifaceted roles throughout the progression of tumorigenesis. Recent evidence suggests that shifts in macrophage metabolic programs—including glycolysis, oxidative phosphorylation, fatty acid utilization, glutamine metabolism, and the pentose phosphate pathway, are closely associated with diverse and context-dependent functional states rather than fixed polarization phenotypes. During tumor progression toward invasion and metastasis, macrophage metabolic programs dynamically adapt to spatial and temporal variations within the TME, often contributing to immunoregulatory or tumor-supportive niches that facilitate angiogenesis, tumor dissemination, immune evasion, and metabolic crosstalk with tumor cells. However, the precise mechanisms underlying these context-dependent adaptations remain incompletely understood.

**Main body:**

This article reviews current evidence regarding TAM activation states and metabolic reprogramming by various signals in the TME during tumorigenesis and tumor progression, as well as dynamic alterations in TAM metabolic patterns. Furthermore, we explore how secondary metabolites present in the TME influence macrophage metabolic reprogramming and summarize current research on potential therapeutic agents targeting macrophage metabolism.

**Conclusions:**

We propose that modulating key metabolic regulators in TAMs or intervening in metabolic-immune crosstalk pathways may offer novel strategies for precision medicine in cancer therapy, providing a theoretical foundation for metabolic intervention-based immunotherapeutic approaches.

## Introduction

Macrophages (Mφ) are a type of innate immune cell that is widely distributed throughout the body. They were first discovered in starfish larvae by Metchnikoff [1, 2]. Macrophages are capable of antigen presentation and tissue repair, and they engulf and destroy tumor cells, senescent cells, bacteria, parasites, and other abnormal cells in the body, thereby contributing to immune defense, homeostasis, and surveillance [3, 4]. Macrophages exhibit remarkable functional plasticity and metabolic adaptability, dynamically responding to environmental cues in a context-dependent manner [5]. Recent studies on macrophages have mainly focused on their origin, metabolism, functional diversity, and dual roles in disease progression and protection.

**Fig. 1:**
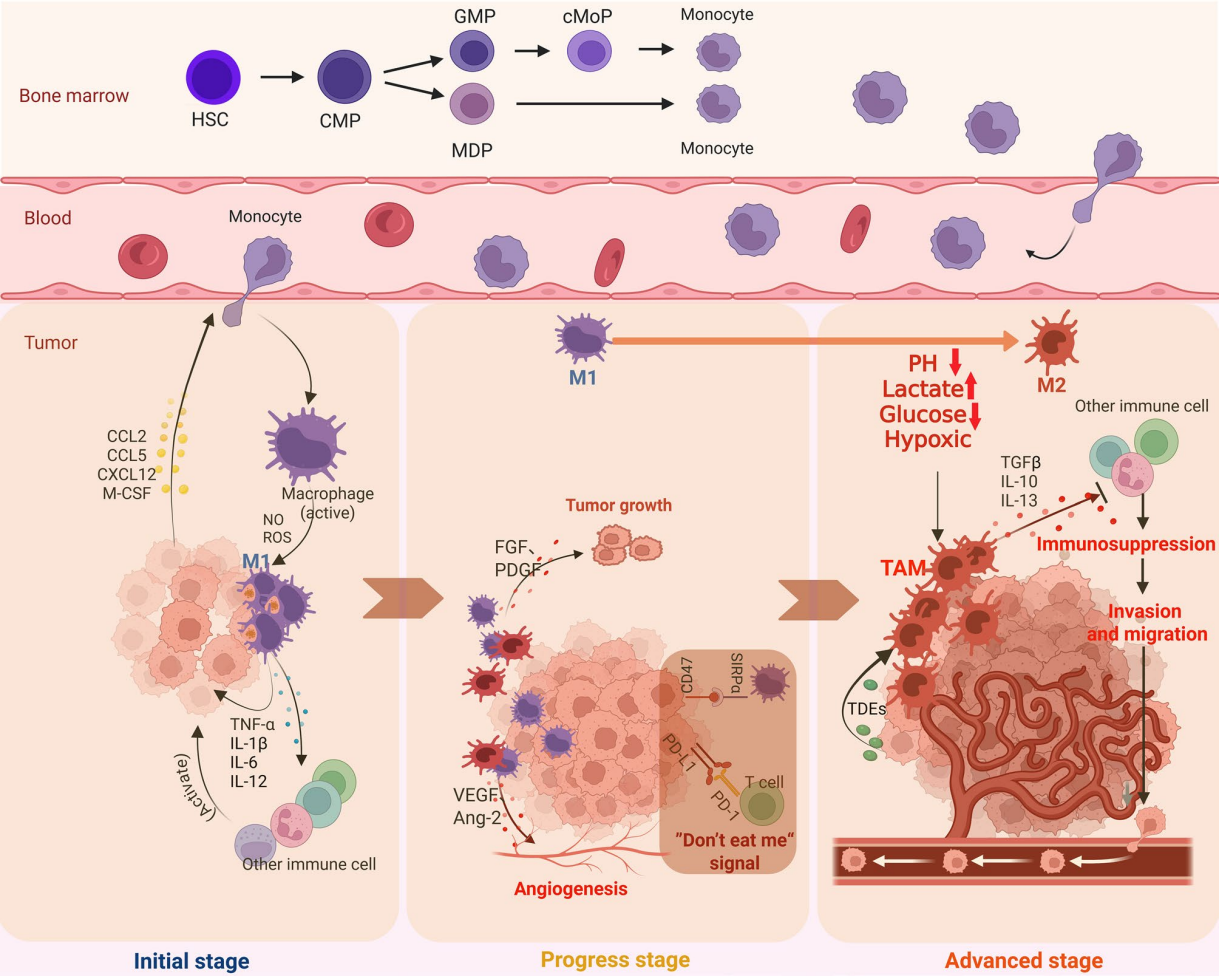
Polarization and symbiosis of macrophages in the development of cancer. In the early stages of tumor development, chemokines secreted by tumor cells facilitate the recruitment of circulating monocytes to the tumor site, where they differentiate into macrophages that may exhibit pro-inflammatory and anti-tumor features. These macrophages can contribute to tumor cell suppression in certain contexts. As tumor burden increases, tumor cells may evade immune surveillance by expressing inhibitory signals, such as “don’t eat me” signals, which modulate macrophage phagocytic capacity. Concurrent metabolic alterations within the tumor microenvironment—including glucose depletion, extracellular acidification, and lactate accumulation—are associated with shifts in macrophage functional programs toward immunoregulatory and tissue-remodeling phenotypes. These macrophage states may support angiogenesis and contribute to the establishment of locally immunosuppressive niches

For many years, the view that tissue macrophages originate from circulating monocytes derived from the bone marrow was widely accepted and has been included in most textbooks [6]. However, the development of stem cell induction models and organoid technology has enabled researchers to reconstruct tissue-like environments in vitro and trace the differentiation and localization of macrophages within these models. These approaches allow the study of how macrophages populate tissues, interact with other cell types, and contribute to inflammation and tumor development, gradually challenging the classical view that all tissue macrophages derive from circulating monocytes [7–9]. Emerging evidence suggests that macrophages in most organs, such as the brain, liver, and kidney, are produced by both the embryonic hematopoietic system and committed hematopoiesis. Among them, the macrophages produced by embryonic hematopoiesis mainly come from the yolk sac and fetal liver hematopoiesis. Some hematopoietic precursor cells differentiate into macrophages during development and colonize tissues. Therefore, they are referred to as tissue-resident macrophages (TRMacs). These tissue-resident macrophages are frequently associated with tissue repair, inflammation resolution, and angiogenesis; however, emerging evidence indicates that their roles in tumor immunity are highly context-dependent and may vary across tumor types and disease stages [10]. On the other hand, macrophages in tissues are also partially derived from bone marrow-directed hematopoiesis, which mainly gives rise to monocytes that further differentiate into macrophages through two bone marrow-derived pathways after birth: 1) Common myeloid progenitor (CMP) → Granulocyte-monocyte progenitor (GMP) → Common monocyte progenitor (cMoP) → Monocyte → Monocyte-derived macrophage; 2) CMP → Monocyte-dendritic cell progenitor (MDP) → Monocyte →Monocyte-derived macrophage. Macrophages generated through these pathways are commonly associated with immune surveillance functions (Fig. 1) [11]. Under inflammatory or oncogenic conditions, circulating monocytes can also be recruited to affected sites through the blood circulation and differentiate into mature monocyte-macrophages, which are capable of rapidly phagocytosing abnormal cells in the body and rely on circulating myeloid monocytes for continuous replenishment (Fig. 1) [1, 11–13].

In general, macrophage development originates from primitive embryonic hematopoiesis and later transitions to definitive hematopoiesis. TRMacs originate from the yolk sac and fetal liver during embryonic hematopoiesis and subsequently colonize various tissues throughout the body during embryonic development. These tissue-resident macrophages are frequently associated with tissue repair and inflammation resolution. However, recent single-cell and spatial transcriptomic studies indicate that their functional states within tumors are highly plastic and shaped by local environmental cues, rather than being strictly determined by developmental origin. Monocyte-derived macrophages are often linked to immune surveillance functions, yet accumulating single-cell and lineage-tracing studies suggest substantial overlap and functional convergence between distinct macrophage populations within tumors [14, 15]. Although the view that macrophages are derived from embryonic hematopoiesis and bone marrow-directed hematopoiesis has been gradually recognized, doubts still remain regarding the specific paths and times, as well as the related regulatory mechanisms for the differentiation of the various precursor cells into TRMacs in different tissues during the embryonic period [16].

During the past fifteen years, with continuous progress in single-cell spatial transcriptome sequencing and extensive studies on macrophage epigenetics, scientists have been able to further analyze the metabolic mechanisms, functional regulation, and related molecular pathways through which macrophages contribute to disease initiation and progression. This review offers an in-depth examination of recent developments in the regulation of macrophage metabolism. We summarize the mechanisms of macrophage activation and polarization during cancer initiation and progression from both spatial and temporal perspectives and delineate the metabolic reprogramming of macrophages throughout these processes. Furthermore, we highlight the regulatory roles of secondary metabolites in the tumor microenvironment (TME) in shaping macrophage metabolic states and compile current therapeutic strategies and potential agents targeting macrophage metabolism. Together, these insights highlight the critical importance and translational potential of metabolic reprogramming in macrophages for disease progression. Importantly, while traditional frameworks such as M1/M2 polarization or glycolytic versus oxidative states have provided conceptual clarity, they may oversimplify the highly dynamic and spectrum-like nature of macrophage states revealed by recent single-cell and spatial omics technologies. In this review, we aim to integrate metabolic insights within a more nuanced and context-aware framework and further discuss the controversies and limitations of current paradigms in the final section.

## Activation and polarization of macrophages in cancer progression

Macrophages respond to a wide range of environmental cues that shape their effector functions in vivo. These signals are frequently mediated through pattern recognition receptors (PRRs), which detect pathogen-associated molecular patterns (PAMPs) and damage-associated molecular patterns (DAMPs) located either on the cell surface or within the intracellular compartments. Major PRR families include Toll-like receptors (TLRs), nucleotide-binding oligomerization domain-like receptors (NLRs), C-type lectin receptors (CLRs), and RIG-I-like receptors (RLRs) [17, 18]. Engagement of these receptors activates interconnected signaling networks that remodel macrophage morphology, transcriptional programs, and metabolic states. Within traditional immunological frameworks, these adaptive changes have often been described as macrophage polarization [19]. Historically, polarized macrophages have been categorized into two broad functional extremes: the pro-inflammatory, classically activated (M1-like) phenotype and the anti-inflammatory, alternatively activated (M2-like) phenotype [20, 21]. However, this binary classification represents a conceptual simplification of a far more heterogeneous and dynamically regulated spectrum of macrophage states observed in vivo.

The TME is established early during tumorigenesis and is characterized by complex interactions among tumor cells, stromal components, and infiltrating immune cells. Tumor- and stroma-derived chemokines, including CCL2, CSF-1, CXCL12, and M-CSF, promote the recruitment of circulating monocytes into tumor sites, where they differentiate into macrophages under the influence of local cues [22, 23]. In certain inflammatory niches, these recruited macrophages may transiently display pro-inflammatory features, including reactive oxygen species (ROS) and nitric oxide (NO) production, as well as secretion of cytokines such as IL-6 and TNF-α. These effector functions can contribute to tumor cell stress and apoptosis in specific contexts [17, 24, 25].

However, macrophage activation states within tumors are highly sensitive to evolving microenvironmental conditions (Fig. 1). As tumors undergo metabolic reprogramming characterized by increased glycolysis, glucose competition, lactate accumulation, and extracellular acidification [26], macrophage metabolic and transcriptional programs are correspondingly reshaped. Rather than deterministically driving a discrete M2 phenotype, lactate and other tumor-derived metabolites have been associated with the emergence of immunoregulatory and tissue-remodeling gene signatures in macrophages [27]. The functional consequences of these signals appear to vary across tumor types and spatial niches.

In addition, tumor-derived exosomes and immune checkpoint ligands, such as PD-L1 and CD47, can modulate macrophage signaling pathways, including NF-κB-dependent mechanisms [23, 28]. These interactions influence macrophage phenotypes in a context-dependent manner and may contribute to the establishment of locally immunosuppressive microenvironments.

As tumor burden increases, macrophages accumulating within the TME—collectively termed tumor-associated macrophages (TAMs)—often exhibit transcriptional programs enriched in angiogenesis, extracellular matrix remodeling, and immunoregulatory pathways. Nevertheless, single-cell analyses have revealed substantial heterogeneity within TAM populations, with coexisting pro-inflammatory, metabolic, and regulatory states rather than a uniform tumor-promoting phenotype [29, 30].

Collectively, these findings highlight the functional diversity of macrophages within tumors. While the M1/M2 framework has historically provided a useful heuristic to describe pro-inflammatory and immunoregulatory tendencies (Fig. 1), accumulating evidence indicates that macrophage states in the TME rarely conform to discrete categories. Instead, single-cell transcriptomic and spatial profiling studies have demonstrated that TAMs exist along a continuum of overlapping transcriptional, metabolic, and functional programs that may coexist within individual cells or micro-niches [31, 32].

Although additional subclassifications, such as M2a–M2d, have been proposed based on in vitro stimulation conditions, their direct correspondence to in vivo macrophage states remains incompletely defined [33]. Thus, contemporary understanding increasingly favors a spectrum-based and context-aware model of macrophage activation, emphasizing dynamic plasticity rather than fixed polarization endpoints.

## Spatial heterogeneity–driven metabolic reprogramming of macrophages during tumor progression and metastasis

The tumor microenvironment is not a homogeneous milieu but rather a complex arrangement of spatially distinct metabolic niches that exert differential pressures on infiltrating macrophages. These spatial metabolic gradients shape macrophage metabolic programs in ways that are not captured by simple linear progression models, underscoring the need to consider both anatomical location and dynamic environmental cues when interpreting macrophage functional states within tumors [34, 35].

### Metabolic niches within Primary tumors: core vs. Invasive margin

Within primary tumors, distinct microanatomical regions, such as hypoxic cores and invasive margins, impose divergent metabolic constraints that drive differential macrophage programming [36]. Tumor cores are often characterized by hypoxia, glucose depletion, lactate accumulation, and extracellular acidification; these conditions collectively promote metabolic adaptations such as enhanced glycolytic flux and HIF‑1α–dependent transcriptional programs in infiltrating macrophages [37]. Concurrently, hypoxic stress upregulates chemokines (e.g., CSF‑1, CCL2) that attract circulating monocytes to the core, where they encounter necrotic debris and stress signals that further influence their metabolic trajectories and functional potential [38, 39].

In contrast, the invasive margin—the interface between tumor and adjacent normal tissue—presents a different metabolic ecology. This region is frequently enriched in oxygen and pro‑inflammatory signals, and harbors resident tissue macrophages that play sentinel roles in detecting tumor invasion. Resident macrophages in the margin display metabolic adaptations, including lipid handling and oxidative metabolism, that are distinct from those in the core [40]. Spatial transcriptomic datasets have identified macrophage subsets in invasive regions with prominent lipid metabolism signatures, including pathways related to unsaturated fatty acid biosynthesis and arachidonic acid metabolism, and elevated expression of lipid receptors such as Trem2 and CD36, consistent with a phenotype resembling lipid‑associated macrophages (LAMs) [41–43].

This metabolic diversity within primary tumors illustrates that macrophage phenotypes cannot be reduced to simplistic axis transitions; rather, they constitute a spatially structured continuum of context‑dependent metabolic programs.

### Metabolic adaptation during tumor dissemination and metastatic colonization

As tumors progress from localized growth to disseminated disease, tumor cells actively remodel distant tissues to form pre‑metastatic niches that facilitate colonization [44, 45]. This systemic remodeling alters local nutrient landscapes and stromal composition, thereby influencing the metabolic phenotypes of infiltrating and resident macrophages at metastatic sites.

During early dissemination, a subset of macrophages may accompany migrating tumor cells through the circulation, encountering unique systemic stresses such as shear forces and oxidative stress. Single‑cell metabolic profiling has demonstrated that these circulating macrophages upregulate antioxidant pathways, including glutathione metabolism, as an adaptive response to maintain viability and support tumor cell survival in transit [46, 47].

Upon arrival at secondary sites, such as liver or lung, the local microenvironment imposes organ‑specific metabolic demands. Tumor cells and stromal elements in metastatic niches can reshape nutrient availability—for instance, high glutamine, lipid, or glucose turnover—which, in turn, directs macrophage metabolic reprogramming toward programs that promote tissue remodeling and immune modulation [48, 49]. In these contexts, macrophages not only adapt their own metabolic states but also engage in reciprocal metabolic interactions with tumor cells. For example, tumor‑derived metabolites such as glutamine are utilized by macrophages to fuel anabolic processes, while macrophage metabolites, including ornithine and polyamines, may support tumor cell proliferation and remodeling of the metastatic niche [40, 50, 51].

Collectively, the spatial dimension of TAM metabolism—from core versus margin within primary tumors to differential metabolic ecologies across metastatic sites—underscores the necessity of integrating anatomical context when interpreting metabolic programs. These spatial metabolic landscapes interact with temporal progression, yielding a multidimensional and dynamic continuum of macrophage functional states.

### Cancer-type– and species-dependent metabolic heterogeneity of TAMs

It should be noted that although highly glycolytic tissues such as fast-twitch muscle produce abundant lactate, tissue-resident macrophages (TRMacs) in these tissues do not undergo the same pro-tumoral metabolic reprogramming as TAMs. This difference likely reflects the absence of tumor-specific signals such as hypoxia, necrosis, inflammatory cytokines, and tissue remodeling cues, which are unique to the tumor microenvironment. In addition to spatial heterogeneity within tumors, TAM metabolic phenotypes vary substantially across cancer types, species, and tumor niches. These differences reflect tumor-intrinsic oncogenic programs, tissue-of-origin metabolic landscapes, oxygen availability, and systemic metabolic states [52].

In highly glycolytic tumors such as breast cancer, elevated lactate production promotes alternative activation programs in macrophages and supports oxidative metabolism and lactate utilization rather than classical glycolysis in TAMs [53, 54]. Tumor-derived lactate can induce macrophage polarization through HIF-1α–dependent signaling and the induction of pro-tumorigenic mediators such as VEGF and Arginase-1(ARG1). Recent studies further demonstrate that lactate-rich tumor microenvironments sustain M2-like macrophage phenotypes and promote immune suppression and tumor progression [53, 55, 56].

In contrast, in desmoplastic and severely hypoxic tumors such as pancreatic ductal adenocarcinoma, TAMs frequently display enhanced fatty acid oxidation (FAO) and mitochondrial respiration, likely driven by nutrient restriction and stromal remodeling within the tumor microenvironment [57, 58]. Increasing evidence suggests that FAO-dependent metabolic programs support the immunosuppressive and pro-tumoral functions of macrophages in several cancers [59, 60].

In metabolically lipid-rich tumors such as hepatocellular carcinoma, macrophages often exhibit lipid accumulation and altered cholesterol metabolism, reflecting the lipid-rich hepatic microenvironment and metabolic crosstalk between hepatocytes and immune cells [61]. In metastatic or nutrient-restricted tumor niches, TAMs may demonstrate increased mitochondrial fitness and oxidative phosphorylation (OXPHOS) dependency in response to metabolic stress within the tumor microenvironment [54, 62]. Recent work further suggests that TAM-derived metabolites such as lactate can actively remodel tumor immunity and influence tumor cell signaling networks [63, 64].

Importantly, species differences further complicate interpretation. Several metabolic features observed in mouse tumor models, including enhanced glycolytic reprogramming or FAO dependence, are not always fully recapitulated in human tumors, highlighting the importance of cross-species validation in immunometabolism studies [65, 66]. Moreover, TAM metabolic states can differ between primary tumors and metastatic niches, reflecting organ-specific nutrient availability, stromal composition, and immune contexture.

These observations underscore that TAM metabolism is not governed by a single universal program but instead represents a context-dependent metabolic adaptation shaped by tumor type, tissue niche, and species-specific physiology. Moreover, it should be noted that many mechanistic insights into TAM metabolic reprogramming have been derived from mouse tumor models and in vitro macrophage polarization systems, whereas direct evidence from human tumors is still emerging and is largely supported by transcriptomic, single-cell, or spatial profiling datasets. To improve interpretability, the context-dependent metabolic heterogeneity of TAMs across cancer types, species, and tumor niches, together with their supporting evidence across human samples and experimental systems, is summarized in Table 1.

**Table 1:** Context-dependent metabolic programs of tumor-associated macrophages across cancer types, model systems, and tumor niches

Metabolic pathway	Tumor type	Tumor niche	Human evidence	Animal / in vitro evidence	TAM functional phenotype	Therapeutic implications	Representative references
Glycolysis	Breast cancer, Lung cancer	Primary tumor	Single-cell RNA-seq and metabolic gene signatures detected in TAMs from patient tumors	Mouse tumor models and in vitro macrophage polarization studies	Enhanced glycolysis supports inflammatory signaling but may also sustain tumor-promoting macrophage activation depending on context	Targeting glycolysis may modulate TAM activation and inflammatory signaling	[53, 67–70]
Lactate metabolism	Melanoma, breast cancer, endometrial cancer	Primary tumor	High lactate levels correlate with M2-like TAM signatures in patient tumors	Lactate-treated macrophages and murine tumor models	Tumor-derived lactate activates HIF-1α signaling and promotes immunosuppressive TAM polarization	Targeting lactate transporters or metabolism may reduce TAM-mediated immunosuppression	[53, 55, 71, 72]
Oxidative phosphorylation (OXPHOS)	Lung cancer, melanoma	Primary tumor and metastasis	Mitochondrial metabolic programs identified in TAM populations by scRNA-seq	Mouse tumor models demonstrating enhanced mitochondrial respiration in TAMs	OXPHOS-dependent TAMs often exhibit immunosuppressive phenotypes supporting tumor growth	Mitochondrial metabolism inhibitors may reprogram TAM function	[62, 73–75]
Fatty acid oxidation (FAO)	Ovarian cancer, breast cancer, colorectal cancer	Primary tumor	Lipid metabolism gene signatures detected in TAMs from patient tumors	Murine tumor models demonstrating FAO-dependent macrophage polarization	FAO supports long-term survival and immunosuppressive TAM phenotypes	FAO inhibitors may reduce tumor-promoting macrophage activity	[74, 76–79]
Lipid uptake and lipid droplet accumulation	Breast cancer, Glioblastoma, ovarian cancer	Primary tumor	Lipid-laden TAMs observed in human tumors	Mouse tumor models showing lipid accumulation in TAMs	Lipid accumulation enhances cytokine production and immunosuppressive signaling	Targeting lipid transport pathways may disrupt TAM-tumor metabolic crosstalk	[80–83]
Glutamine metabolism	Hepatocellular carcinoma, pancreatic cancer	Primary tumor	Glutamine metabolic signatures observed in TAM transcriptomic analyses	Macrophage metabolic inhibition experiments and murine tumor models	Glutamine utilization supports TAM survival and pro-tumoral polarization	Targeting glutamine metabolism may disrupt TAM-mediated tumor support	[84–87]
Arginine metabolism (ARG1 pathway)	Glioblastoma, breast cancer, pancreatic cancer	Primary tumor	ARG1-expressing TAMs detected in human tumors	Mouse models demonstrating ARG1-dependent suppression of T-cell responses	Arginine depletion suppresses antitumor T-cell activity	Arginase inhibitors are being explored for immunotherapy	[88–92]
Tryptophan metabolism (IDO pathway)	Glioblastoma, lung cancer	Primary tumor and metastasis	IDO expression detected in TAMs from patient tumors	Mouse tumor models and pharmacological inhibition studies	IDO activity promotes T-cell exhaustion and immune tolerance	IDO inhibitors are being investigated in cancer immunotherapy	[93–96]
Succinate signaling and TCA remodeling	Lung cancer	Primary tumor	Metabolic profiling suggests altered TCA activity in TAM populations	Genetic mouse models affecting macrophage metabolic enzymes	Succinate signaling regulates inflammatory and epigenetic programs	Targeting TCA intermediates may modulate macrophage activation	[97–100]
Ketone body metabolism	Hepatocellular carcinoma	Primary tumor	Ketone metabolism enzymes detected in TAM transcriptomes	Mouse tumor models manipulating ketolysis pathways	Ketone metabolism influences macrophage polarization and T-cell exhaustion	Ketone metabolic pathways may represent emerging therapeutic targets	[101]
Cholesterol metabolism	Gastric cancer	Primary tumor	Cholesterol metabolism genes enriched in TAM populations from patient tumors	Mouse tumor models targeting CH25H	Cholesterol accumulation promotes immunosuppressive macrophage phenotypes	Modulating cholesterol metabolism may restore antitumor immunity	[102–104]

## Metabolic pattern transformation of macrophages during cancer development and progression

### Metabolic reprogramming of macrophages during tumor progression

During cancer progression, macrophages exhibit distinct yet context-dependent metabolic patterns that are often associated with different polarization states, enabling them to adapt to the TME. Classically activated (M1-like) macrophages, exhibiting pro-inflammatory and anti-tumor functions, are generally characterized by increased glycolysis and pentose phosphate pathway activity. In contrast, alternatively activated (M2-like) macrophages, which possess anti-inflammatory and pro-tumor properties, more frequently display enhanced oxidative phosphorylation (OXPHOS), glutamine and fatty acid oxidation (FAO), and polyamine metabolism [105–107]. However, emerging evidence suggests that these metabolic features may overlap in vivo and do not represent strictly dichotomous states.

**Fig. 2:**
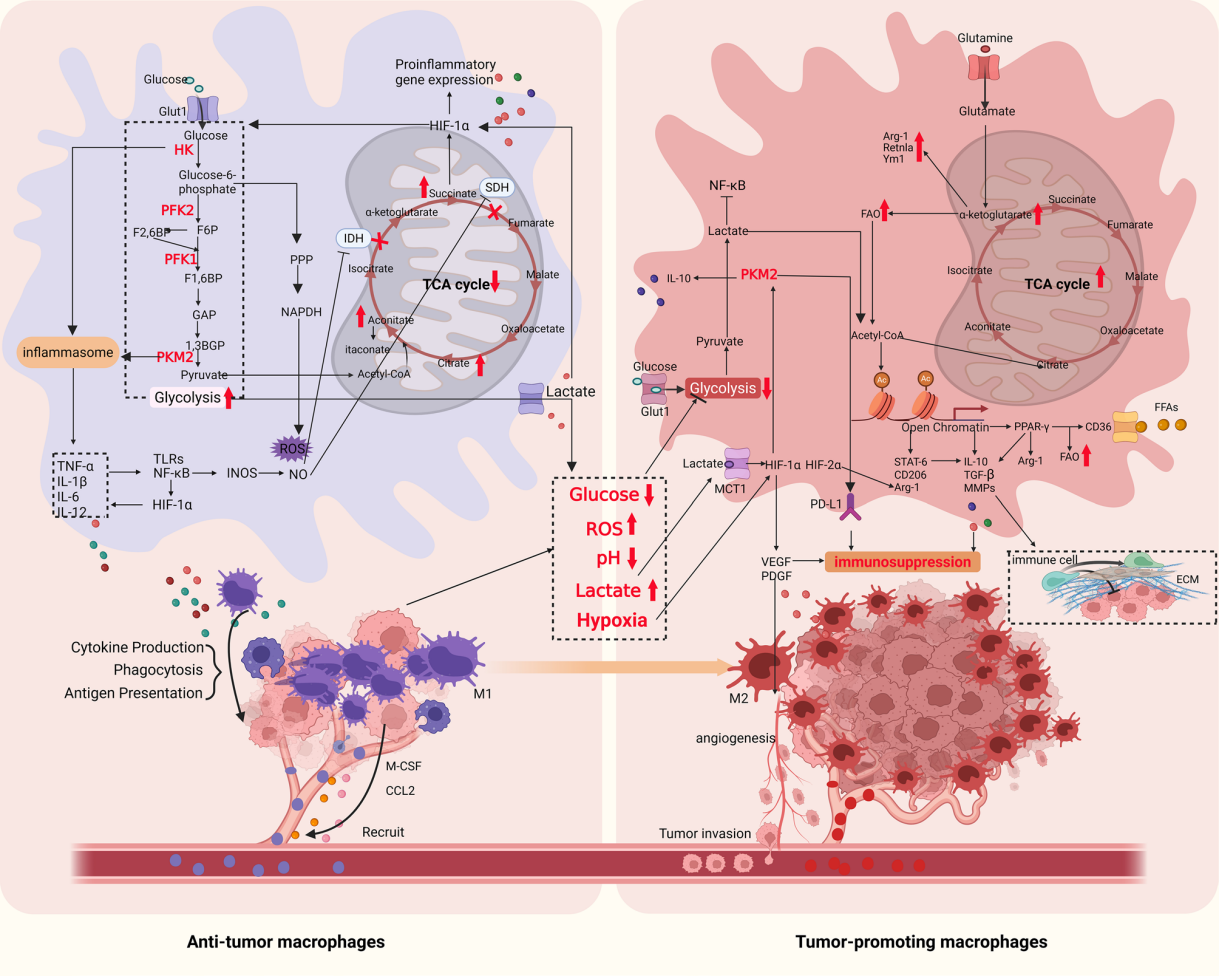
Metabolic pattern transformation of macrophages in the development of cancer. **a**. In early tumor-associated inflammatory contexts, subsets of macrophages exhibiting pro-inflammatory and anti-tumor features are frequently characterized by elevated aerobic glycolysis. Glycolysis-associated metabolic intermediates can interact with TLR and NF-κB signaling pathways, contributing to increased iNOS expression and the production of nitric oxide (NO) and reactive oxygen species (ROS), which may impose stress on tumor cells in specific microenvironmental niches. Elevated NO and ROS levels can also influence mitochondrial function, modulating the catalytic activity of enzymes such as IDH and SDH and thereby reshaping TCA cycle flux. **b**. As the tumor microenvironment undergoes progressive metabolic remodeling—including glucose competition, hypoxia, lactate accumulation, and extracellular acidification—macrophage metabolic programs are dynamically reconfigured. Rather than representing a uniform switch, these conditions are associated with increased reliance on oxidative phosphorylation, fatty acid oxidation, and glutamine metabolism in certain tumor-associated macrophage subsets. Such metabolic adaptations often coexist with transcriptional programs enriched in immunoregulatory and tissue-remodeling pathways, which may contribute to angiogenesis and local immune modulation

In the early stages of tumor development, macrophages with M1-like pro-inflammatory features are frequently observed in the tumor microenvironment. These cells typically increase glucose uptake and generate adenosine triphosphate (ATP) through aerobic glycolysis to meet their high energy demands [108, 109], although such metabolic characteristics may vary depending on tumor type and local microenvironmental conditions. Several critical glycolytic rate-limiting enzymes have been demonstrated to regulate the secretion of inflammatory factors by macrophages. Hexokinase (HK) catalyzes the phosphorylation of glucose to glucose-6-phosphate (G6P), which initiates the glycolytic process. Studies have shown that HK1 can promote the inactivation of glyceraldehyde-3-phosphate dehydrogenase (GAPDH), thereby contributing to inflammasome assembly and activation, which results in the production of pro-inflammatory cytokines, including IL-1β and IL-6 [110, 111]. Phosphofructokinase 1 (PFK1), a key rate-limiting enzyme in the glycolytic pathway, is critical for glycolysis. Studies have indicated that zinc finger and homeobox 2 (ZHX2) binds to the promoter region of PFKFB3 (also known as PFK2) and enhances its transcription, thereby promoting glycolysis. ZHX2 deficiency markedly attenuated the production of IL-6 and IL-1β by M1 macrophages [112]. Pyruvate kinase (PK) functions as a major rate-limiting enzyme in glycolysis, facilitating the conversion of phosphoenolpyruvate (PEP) to pyruvate. The pyruvate kinase M2 isoform (PKM2) not only induces the expression of pro-IL-1β but also facilitates its activation via inflammasomes [113]. Additionally, pro-inflammatory cytokines released by M1 macrophages stimulate TLRs and activate NF-κB signaling pathways, further amplifying the inflammatory response and upregulating inducible nitric oxide synthase (iNOS). This leads to arginine breakdown, resulting in increased production of NO and ROS, which have been shown to exert tumor-inhibitory effects in certain contexts [114–117]. Moreover, TLR and NF-κB signaling contribute to the stabilization of hypoxia-inducible factor HIF-1α, thereby reinforcing M1 polarization [118, 119]. These results underscore the important role of glycolytic metabolism in pro-inflammatory macrophage responses during the early phase of tumor formation (Fig. 2), while recognizing that alternative metabolic adaptations may coexist in certain contexts. During glycolysis, M1 macrophages engage in the pentose phosphate pathway (PPP). NADPH produced by the PPP promotes the synthesis of reduced glutathione, enabling the timely clearance of intracellular ROS and thereby maintaining the survival of inflammatory macrophages [120]. Additionally, enhanced arginine–succinate metabolism in M1 macrophages contributes to the regeneration of arginine, fumarate, and malate. The generation of NO and ROS from arginine metabolism disrupts mitochondrial function and inhibits the catalytic activities of isocitrate dehydrogenase (IDH) and succinate dehydrogenase (SDH) [121, 122]. Furthermore, under conditions of local hypoxia within the TME, HIF-1α becomes activated, promoting inflammatory cytokine secretion and preventing pyruvate entry into mitochondria, thereby partially restraining the tricarboxylic acid (TCA) cycle [123].

As tumors continue to progress, cancer cells often exhibit enhanced glycolysis, consuming substantial amounts of glucose and contributing to hallmark features of the TME, including glucose deprivation, hypoxia, and lactic acid accumulation [26, 124]. These environmental changes are thought to contribute to shifts in macrophage metabolic profiles, although the extent and direction of such transitions may vary across tumor contexts. In glucose-deprived regions of the TME, glycolysis-dependent metabolic activity in M1-like macrophages may be attenuated, potentially impairing their effector functions and favoring the emergence of M2-like features. Macrophages exhibiting M2-like functional properties often rely more heavily on OXPHOS, FAO, and glutamine metabolism, and are typically associated with a relatively intact TCA cycle. Notably, the accumulation of α-ketoglutarate (α-KG) has been reported to function as an important metabolic signal that promotes FAO and the expression of M2 markers such as ARG1 through epigenetic and signaling mechanisms, ultimately reinforcing the immunosuppressive and tissue-repair functions of M2 macrophages [105, 125]. Low-glucose conditions within the TME have been reported to promote CD36 upregulation in certain macrophage subsets, increase fatty acid uptake, markedly enhance intracellular lipid droplet formation, and induce the polarization of TAMs toward the M2 phenotype via activation of the STAT6 signaling pathway [126, 127]. The increased uptake of fatty acids further upregulates peroxisome proliferator-activated receptor gamma (PPAR-γ) expression. [128, 129]. PPAR-γ is a key transcription factor regulating cellular lipid metabolism and has been implicated in macrophage polarization. By promoting FAO and mitochondrial biogenesis, PPAR-γ maintains metabolic homeostasis and, in cooperation with its coactivator PGC-1β, directly drives the transcription of M2-associated genes such as ARG1. This regulatory axis reinforces the immunosuppressive and tissue repair capacities of M2 macrophages [130]. Enhanced FAO has been shown to activate STAT-3/STAT-6 signaling pathways in certain settings, upregulates OXPHOS-related genes, suppresses glycolysis, and supports the survival and functional integrity of M2 macrophages [131, 132]. Acetyl-CoA generated from fatty acid oxidation not only enters the TCA cycle to supply cellular energy but also serves as an acetyl donor for histone acetylation, thereby modulating the expression of M2-associated transcription factors such as STAT6 and PPAR-γ. Consequently, it may contribute to the regulation of immunomodulatory cytokine secretion, including TGF-β, IL-10, and matrix metalloproteinases (MMPs), contributing to the establishment of an immunosuppressive microenvironment (Fig. 2) [133].

Although multiple studies have suggested that M2-like macrophages more frequently rely on OXPHOS and FAO while exhibiting comparatively reduced glycolytic activity, glycolysis may still play a supportive role in M2 polarization. For instance, the inhibition of glycolysis using 2-deoxyglucose (2-DG) has been reported to suppress the expression of M2-associated genes such as *Arg1*, *Retnla*, *Mgl2*, and *Mrc1* (CD206), suggesting that glycolytic flux may contribute to certain aspects of M2-like polarization [134, 135]. Pyruvate kinase M2 (PKM2), a key glycolytic enzyme, has been reported to regulate macrophages with M2-like features, primarily observed in in vitro polarized M2 macrophages (Fig. 2). PKM2 promotes the accumulation of glycolytic intermediates and extracellular ATP hydrolysis, enhancing adenosine production and activating the adenosine A2A receptor (A2aR). Downstream signaling via the cAMP–PKA–CREB and STAT3 pathways subsequently increases IL-10 expression, reinforcing an immunosuppressive phenotype. Whether this PKM2–A2aR–IL-10 axis operates in TAMs in vivo and across different tumor types remains to be determined [136–139]. Additionally, PKM2 has been implicated in the upregulation of PD-L1 expression in macrophages, which may further contribute to the establishment of an immunosuppressive TME and facilitate tumor immune evasion [140, 141].

Hypoxia is another prominent feature of the TME. Studies suggest that acute and chronic hypoxia exert partially distinct biological effects. Acute hypoxia preferentially stabilizes HIF-1α, which regulates the transcription of glycolysis-related genes, whereas chronic hypoxia has been associated with increased HIF-2α activity that may control genes involved in FAO and angiogenesis [142–146]. In macrophages, these differential HIF signaling programs have been linked to distinct functional states, with HIF-1α generally associated with pro-inflammatory (M1-like) responses and HIF-2α linked to more immunoregulatory phenotypes. However, the balance between HIF-1α and HIF-2α signaling may vary depending on tumor type, oxygen gradients, and spatial niches within the TME. For example, upon nuclear translocation, HIF-2α can induce the expression of arginine metabolism-related genes such as ARG1, thereby contributing to the acquisition of M2-like functional features [147, 148]. Additional studies have indicated that HIF-1α and HIF-2α may exert partially opposing effects in the regulation of NO production by macrophages. HIF-1α induction has been shown to stimulate iNOS expression and NO synthesis, whereas HIF-2α activation has been associated with increased ARG1 expression and relative suppression of NO production. Nevertheless, emerging data suggest that these regulatory interactions are context-dependent and may not represent a strictly antagonistic relationship in all tumor settings. The differential modulation of HIF-1α and HIF-2α under varying degrees of hypoxia may represent a critical mechanism underlying the functional divergence of macrophage subtypes [143, 147]. Moreover, accumulating evidence suggests that lactate accumulation in the TME can stabilize HIF-1α, potentially promoting angiogenesis and immunosuppressive signaling and modulating the transcription of anti-inflammatory genes such as IL-10 and IL-13 [141, 149, 150]. However, despite increased angiogenic signaling during tumor progression, tumor vasculature is typically abnormal and inefficient. Disorganized, tortuous, and highly permeable vessels lead to irregular blood flow and impaired nutrient delivery, resulting in persistent hypoxia and nutrient deprivation in the TME [151]. Thus, these findings underscore the complex and context-dependent regulatory roles of HIF-1α- and HIF-2α-mediated hypoxic signaling in shaping macrophage responses and modulating downstream gene expression within the TME.

These studies collectively illustrate dynamic changes in the TME during tumor progression, which are frequently accompanied by shifts in macrophage metabolic programs from relatively anti-tumor to more pro-tumor functional states (Fig. 2). However, such stage-oriented descriptions represent a simplified conceptual framework rather than a strictly linear biological process. Increasing evidence, particularly from single-cell transcriptomic and spatial analyses, suggests that tumor-associated macrophages exist along a metabolic and functional continuum shaped by local microenvironmental cues [31, 152]. Furthermore, current TAM-related studies in patients with clinical tumors remain relatively limited. The upstream triggering mechanisms governing macrophage metabolic regulation are not fully defined, and several aspects of glycolytic involvement in M2-like polarization require further mechanistic clarification.

### Contextual determinants of macrophage metabolic reprogramming: physiological glycolysis versus tumor metabolic stress

Although enhanced glycolysis is frequently associated with immunometabolic reprogramming in tumors, glycolytic activity per se is insufficient to explain the distinct functional states of macrophages or tissue-resident memory T cells (TRM) within the tumor microenvironment (TME) [67, 153, 154]. Certain physiological tissues, such as fast-twitch skeletal muscle, exhibit high glycolytic flux under normal conditions; however, they do not induce persistent immunosuppression or the metabolic exhaustion observed in tumors [155, 156].

This discrepancy highlights that metabolic intensity alone does not determine immune cell fate [154]. In physiological settings, glycolytic bursts are typically transient, tightly regulated, and not accompanied by sustained inflammatory signaling or nutrient deprivation [157]. In contrast, the TME is characterized by chronic hypoxia, continuous lactate accumulation, prolonged glucose competition, acidic pH, and persistent exposure to tumor-derived cytokines such as TGF-β and IL-10 [54, 158]. These factors collectively establish a long-term pathological metabolic niche that reshapes macrophage bioenergetics and signaling networks.

Importantly, metabolic stress in tumors is spatially heterogeneous and temporally sustained, resulting in stable transcriptional and epigenetic reprogramming [65]. By comparison, metabolic fluctuations in normal tissues are reversible and homeostatically buffered. Thus, it is the integration of metabolic stress with oncogenic signaling and inflammatory cues—rather than glycolysis alone—that drives durable immunometabolic reprogramming in TAMs [153, 159].

These observations underscore the necessity of considering metabolic context when interpreting macrophage phenotypes, particularly when extrapolating findings from highly glycolytic physiological tissues to tumor settings.

## Regulatory effects of metabolic intermediates on macrophage metabolism

During tumor development, close bidirectional regulation exists between the TME and macrophage metabolic reprogramming. This interaction is considered one of the key mechanisms associated with tumor growth, invasion, and metastasis and represents an important factor influencing macrophage metabolic adaptation [160]. In recent years, many studies have shown that in the TME, metabolic intermediates of macrophages and tumor cells participate in macrophage metabolic reprogramming and anti-tumor responses by modulating cell signal transduction and gene expression. These regulatory mechanisms include direct interaction with signaling pathway protein components, regulation of metabolite sensor pathways, influence on protein stability or activity, and regulation of the epigenome and epitranscriptome. In addition, some metabolic byproducts can be released into the TME in a paracrine manner, transmitting signals to neighboring cells, thereby mediating intercellular communication [141]. Emerging evidence from single-cell and spatial analyses further suggests that such metabolic crosstalk is highly heterogeneous and context-dependent rather than uniformly regulated across all macrophage subsets [61, 161].

### Lactic acid

To meet the energy needs of rapid proliferation after cell carcinogenesis, cancer cells often exhibit increased glycolytic activity. Even under aerobic conditions, cancer cells can produce lactic acid and ATP through aerobic glycolysis, a phenomenon known as the Warburg effect [27, 162].

Glucose serves as a major substrate for energy metabolism in tumors and immune cells during the initial stages of carcinogenesis and tumor progression. The metabolism of tumor cells and polarized M1 macrophages (anti-tumor phenotype) is often characterized by elevated glycolytic activity, resulting in the gradual production of substantial amounts of lactic acid in the TME, accompanied by a decrease in pH (pH 6.0–6.5) [27]. As mentioned above, as the tumor continues to expand, cancer cells and immune cells, such as macrophages, compete for metabolic substrates through the glycolytic pathway, leading to the gradual accumulation of lactic acid in the TME. Rather than uniformly forcing macrophages to switch metabolic programs, elevated lactate levels are thought to modulate macrophage functional states in a context-dependent manner, potentially favoring immunoregulatory or tissue-remodeling phenotypes in certain microenvironmental settings [163]. During these physiological processes, HIF-1α plays an important role in the regulation of glycolysis and lactate production [71, 164]. Studies have shown that lactate in the TME can enter macrophages via monocarboxylate transporters (MCT1/MCT4) and is converted to pyruvate by lactate dehydrogenase. Subsequently, the pyruvate dehydrogenase complex catalyzes the oxidative decarboxylation of pyruvate to generate acetyl-CoA, which enters the mitochondrial TCA cycle and may contribute to OXPHOS activity in certain macrophage subsets, potentially influencing their functional polarization in a context-dependent manner [136, 165]. Lactate in the TME can be sensed by GPR81 on the macrophage membrane, which modulates intracellular signaling pathways and has been associated with attenuation of NF-κB activation. Emerging evidence suggests that this process may involve the inactivation of YAP/TAZ, thereby contributing to the acquisition of immunoregulatory features in macrophages [166]. It has been reported that lactic acid is also involved in the epigenetic reprogramming of macrophages. Enhanced acetylation of histone H3K27 has been associated with altered chromatin accessibility at multiple inflammatory gene loci, reduced secretion of pro-inflammatory factors such as IL-6 by macrophages, indirect inhibition of antigen presentation, and increased transcription of markers commonly associated with M2-like phenotypes (such as ARG1 and CD206) [149, 167]. Nevertheless, single-cell transcriptomic analyses suggest that these epigenetic and transcriptional features may not be uniformly distributed across all tumor-associated macrophage populations [168, 169]. On the other hand, lactate in macrophages can also activate the STAT-3/STAT-6 pathway through histone acetylation, induce IL-10 and TGF-β secretion, inhibit CD8^+^ T cell function, and promote tumor angiogenesis in certain contexts [53]. In addition, lactic acid in macrophages can stabilize the expression of HIF-1α and HIF-2α by inhibiting the activity of PHDs, thereby regulating anti-inflammatory and tissue repair processes (Fig. 2) [53, 170, 171].

These studies collectively suggest that lactic acid participates in macrophage metabolic remodeling through integrated mechanisms involving metabolic substrate availability, microenvironmental acidification, signaling pathway modulation, and epigenetic regulation. Rather than driving a strictly linear transition from “glycolytic M1” to “OXPHOS-dependent M2” states, lactate appears to influence the balance between glycolytic and oxidative metabolic programs in a context-dependent manner [172]. Additionally, it has been associated with the emergence of anti-inflammatory and immunoregulatory features in certain macrophage subsets. The transformation of macrophage metabolic patterns is increasingly recognized as a contributing factor in tumor immune escape, angiogenesis, and tumor cell invasion and metastatic dissemination, although these effects may vary across tumor types and microenvironmental contexts.

### Arginine

Arginine is a conditionally essential amino acid that is mainly derived from dietary intake and intestinal–renal metabolic pathways. Normal cells consume arginine primarily through cationic amino acid transporters (CAT1-3) in a Na^+^ -independent manner [173]. During macrophage metabolism, arginine utilization is largely mediated by iNOS and ARG1, which are closely associated with macrophage functional states and metabolic programs rather than strictly defined polarization phenotypes [174].

**Fig. 3:**
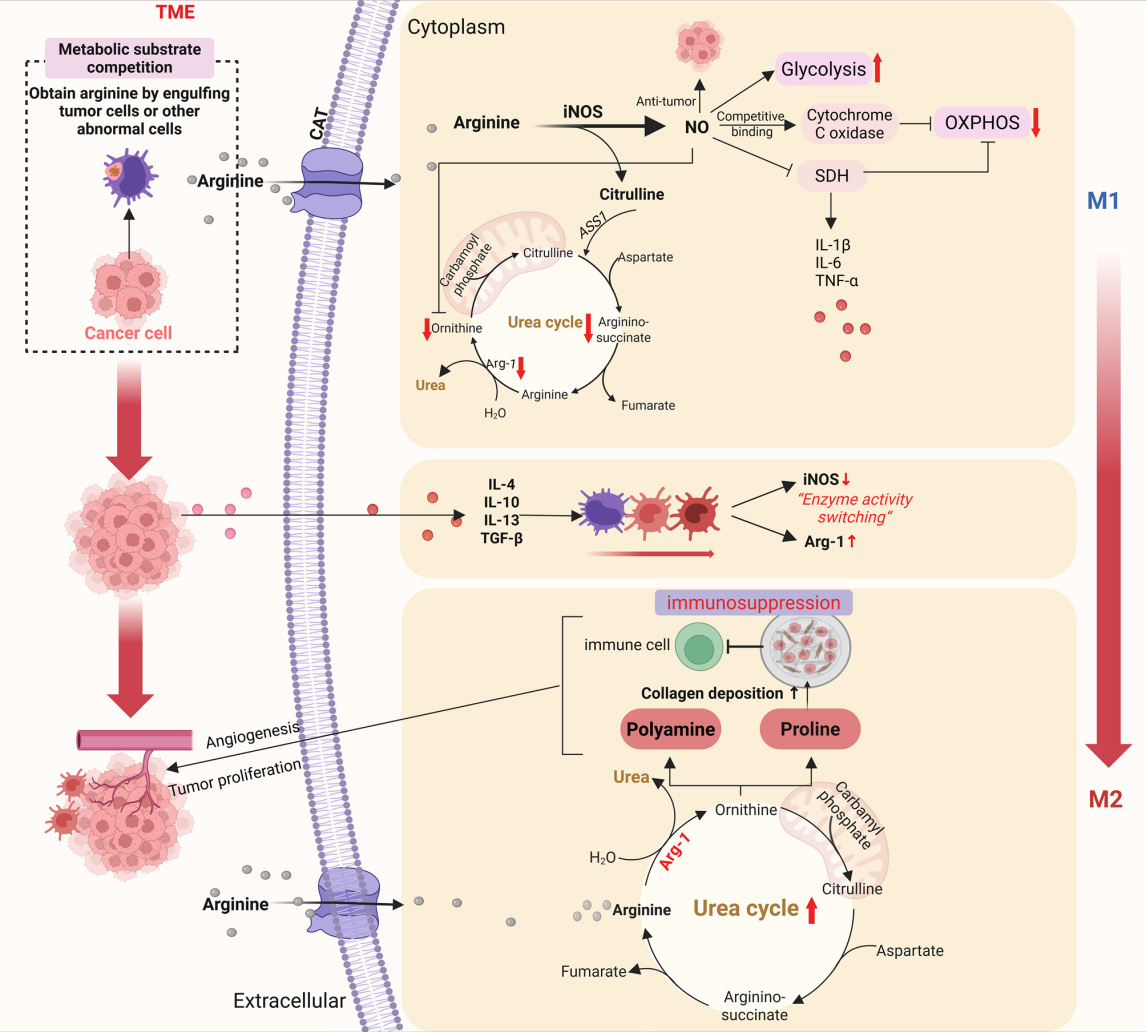
Arginine metabolic alterations in macrophages during cancer progression. In inflammatory tumor niches, subsets of macrophages with pro-inflammatory characteristics display increased arginine uptake and iNOS-associated NO production, accompanied by variable levels of citrulline generation. As tumor progression is accompanied by evolving immunoregulatory conditions, tumor-associated macrophages (TAMs) frequently exhibit altered arginine metabolism, characterized by reduced iNOS expression and enhanced ARG1 expression in specific subpopulations. ARG1 catalyzes the conversion of arginine to ornithine and urea, thereby contributing to urea cycle–related metabolic pathways. Downstream metabolites of ornithine, including polyamines and proline, are associated with tissue remodeling, cell proliferation, and extracellular matrix dynamics within the tumor microenvironment. These metabolic programs may coexist with other functional states rather than representing mutually exclusive polarization endpoints

In the early stages of cancer, cancer cell proliferation requires a large amount of energy and metabolic substrates, and shows a strong dependence on arginine to meet metabolic needs, such as polyamine, protein, and NO synthesis [175]. At this time, macrophages with anti-tumor functional features can compete with tumor cells for arginine in the TME or obtain arginine by engulfing tumor cells and other abnormal cells. Simultaneously, high iNOS expression catalyzes arginine to produce high concentrations of NO along with smaller amounts of citrulline [176]. NO serves as a versatile signaling molecule, exerting both anti-tumor and immunomodulatory effects [177, 178]. In pro-inflammatory macrophages, NO competes with oxygen for binding to cytochrome c oxidase (complex IV), thereby inhibiting the electron transport chain and promoting a shift toward glycolysis to meet cellular energy demands [179]. In addition, NO can further suppress oxidative phosphorylation by reducing the activity of TCA cycle enzymes such as IDH and SDH (Fig. 3) [180–183]. High iNOS expression also competes with ARG1 for arginine, partially inhibiting the urea cycle and reducing ornithine production, which reinforces macrophages’ pro-inflammatory functional state and supports inflammatory responses [184]. Peng et al. discovered that citrulline, generated by iNOS during arginine metabolism, also has an important effect on macrophage function. Under normal conditions, citrulline produced in pro-inflammatory macrophages is rapidly metabolized by argininosuccinate synthase 1 (ASS1). However, when citrulline accumulates in macrophages, it can directly interact with JAK2, weakening JAK2 binding to IFNγR2 and STAT1, and thereby attenuating JAK2–STAT1 signaling and inhibiting the pro-inflammatory polarization of macrophages [122]. In addition, the functional outcomes of NO in macrophages appear to be concentration-dependent, which may be related to the concentration of NO in the TME. As tumors expand, reduced iNOS expression and decreased NO production in the TME may contribute to shifts in macrophage functional states. Low concentrations of NO inhibit the differentiation of vascular stem/progenitor cells into osteoblasts and promote differentiation toward vascular endothelial cells via modulation of the TGF-β/SMAD2/3 pathway [185–188]. Concurrently, with tumor progression and declining iNOS expression, cancer cells increasingly secrete anti-inflammatory cytokines such as TGF-β and IL-4, which bias macrophages toward tissue-repairing and immunoregulatory functional states, while upregulating ARG1 and reducing iNOS activity. ARG1 catalyzes the conversion of arginine into ornithine and urea. Ornithine serves as a precursor for polyamines (putrescine, spermidine) and proline, in which polyamines can promote cell proliferation and tissue repair [189, 190], and proline is involved in collagen synthesis, fibrosis, and inflammation elimination [191, 192]. These metabolic products further bias macrophages toward immunoregulatory, tissue-repairing phenotypes [184, 193]. In the late stages of tumor development, tumor-promoting macrophages produce the above metabolites through ARG1 decomposition of arginine to regulate neovascularization, tumor cell invasion, and migration. Simultaneously, this leads to excessive collagen deposition, promotes tissue fibrosis, impedes CD8^+^ T cell infiltration into tumors, and ultimately contributes to the establishment of an immunosuppressive environment (Fig. 3) [193].

Arginine exerts a dynamic influence on macrophage functional states, energy metabolism, and immune function through its metabolites (e.g., NO and polyamines), related enzymes, and competitive metabolic pathways, underscoring its central role in shaping macrophage metabolic programs and functional plasticity within the TME. (Fig. 3).

### Glutamine

Glutamine is a conditionally essential amino acid that supports multiple cellular processes, including energy production and biosynthesis. The concentration of glutamine in plasma is approximately 500–1000 μmol/L, making it the most abundant amino acid in human blood [194]. Glutamine enters cells primarily through the amino acid transporter ASCT2/SLC1A5. Part of the intracellular glutamine pool is used for the synthesis of glucosamine, nucleotides, and asparagine, and can also activate the mTORC1 signaling pathway [195]. The remaining glutamine, once transported into mitochondria, is converted into glutamate via a deamination reaction catalyzed by glutaminase (GLS). Glutamate is converted to α-KG by glutamate dehydrogenase (GDH, encoded by GLUD1) or aspartate aminotransferase (AST, encoded by GOT1/GOT2) and participates in the TCA cycle to provide energy and anabolic precursors for cells [196].

The availability of glutamine in the TME is shaped by the combined contributions of tumor cell metabolism, stromal cell secretion, blood circulation, and the broader metabolic network within the microenvironment. In the early stages of tumor development, tumor cell density remains low, and glutamine is primarily derived from blood circulation or local tissue synthesis. As tumors expand, increasing numbers of tumor cells enhance glutamine uptake and catabolism in the TME, facilitated by elevated expression of SLC1A5 and metabolic enzymes such as glutaminase 1 (GLS1) [197]. Concurrently, immune cells (such as macrophages) and stromal cells (such as cancer-associated fibroblasts, CAFs) in the TME competitively consume glutamine, further exacerbating its local deficiency [198]. In the early stages of tumorigenesis, macrophages in the TME predominantly exhibit pro-inflammatory functional features, with energy metabolism largely relying on aerobic glycolysis and a relatively low dependence on glutamine. However, exogenous glutamine not only serves as a nutrient substrate but also mediates specific signals through the amino acid transport pathway, activating the PI3K/Akt-mTORC1 axis [199–201]. Studies have shown that glutamine uptake by macrophages triggers critical signaling cascades, including Akt/PKB and mTORC1. mTORC1 can further activate HIF-1α, facilitating the secretion of pro-inflammatory cytokines and biasing macrophages toward pro-inflammatory functional states in a context-dependent manner [202, 203]. Glutamine deprivation attenuates LPS-induced expression of IL-1β and HIF-1α in macrophages [204]. These findings indicate that macrophages exhibiting pro-inflammatory functional states rely on glutamine metabolism for optimal activity. On the other hand, glutamine taken up by macrophages exhibiting pro-inflammatory functional features is metabolized by enzymes such as GLS and GDH to generate glutamate, arginine, aspartate, and α-KG [205]. In particular, glutamate can be used for glutathione synthesis, which mitigates ROS-induced cellular damage and maintains redox homeostasis in macrophages with pro-inflammatory features [206, 207]. Arginine contributes to NO production in macrophages exhibiting pro-inflammatory functional states, which can support anti-tumor activities [176, 177]. Aspartate can facilitate the purine nucleotide cycle, alleviate cytoplasmic acidification, and enhance activation of HIF-1α and inflammasome signaling in macrophages exhibiting pro-inflammatory functional features [208, 209]. Since NO inhibits IDH and SDH activities, the α-KG generated from glutamine metabolism may not fully drive the TCA cycle. However, α-KG represents a central metabolic hub and can be rapidly utilized in multiple metabolic processes, including amino acid metabolism and epigenetic regulation. Consequently, these metabolic alterations may lead to intracellular succinate accumulation and a reduced α-KG/succinate ratio, which stabilizes HIF-1α and promotes the production of pro-inflammatory cytokines such as IL-1β [105, 205]. In summary, during the early stages of tumor development, although glutamine metabolism is not the dominant metabolic pathway for macrophages supporting anti-tumor functions, glutamine intake contributes to redox homeostasis and the secretion of inflammatory factors in macrophages exhibiting pro-inflammatory functional features.

When a tumor develops to a later stage, glutamine metabolism can become a critical alternative pathway for macrophages under nutrient-limited conditions. Glutamine uptake and subsequent metabolism generate substantial amounts of α-KG, which can supply NADH and FADH2 to support OXPHOS and drive the TCA cycle in certain macrophage subsets [125]. An increased α-KG/succinate ratio can bias macrophages toward tissue-repairing and immunoregulatory functional states [105]. α-KG acts as a cofactor for TET DNA demethylases and the histone demethylase Jmjd3. TET enzymes catalyze the oxidation of 5mC to promote DNA demethylation, while Jmjd3 removes the repressive H3K27me3 mark. Together, these epigenetic modifications can enhance the transcription of genes associated with anti-inflammatory and tissue-repairing functions [210]. Conversely, α-KG generated from glutamine metabolism can inhibit protein kinase IKKβ through a PHD-dependent mechanism, thereby downregulating the NF-κB pathway and limiting activation of macrophages exhibiting pro-inflammatory functional features [105]. At the same time, α-KG can support shifts in macrophage metabolic profiles toward FAO-dominated and tissue-repairing functional states by modulating the expression of FAO-related proteins [105, 211].

**Fig. 4:**
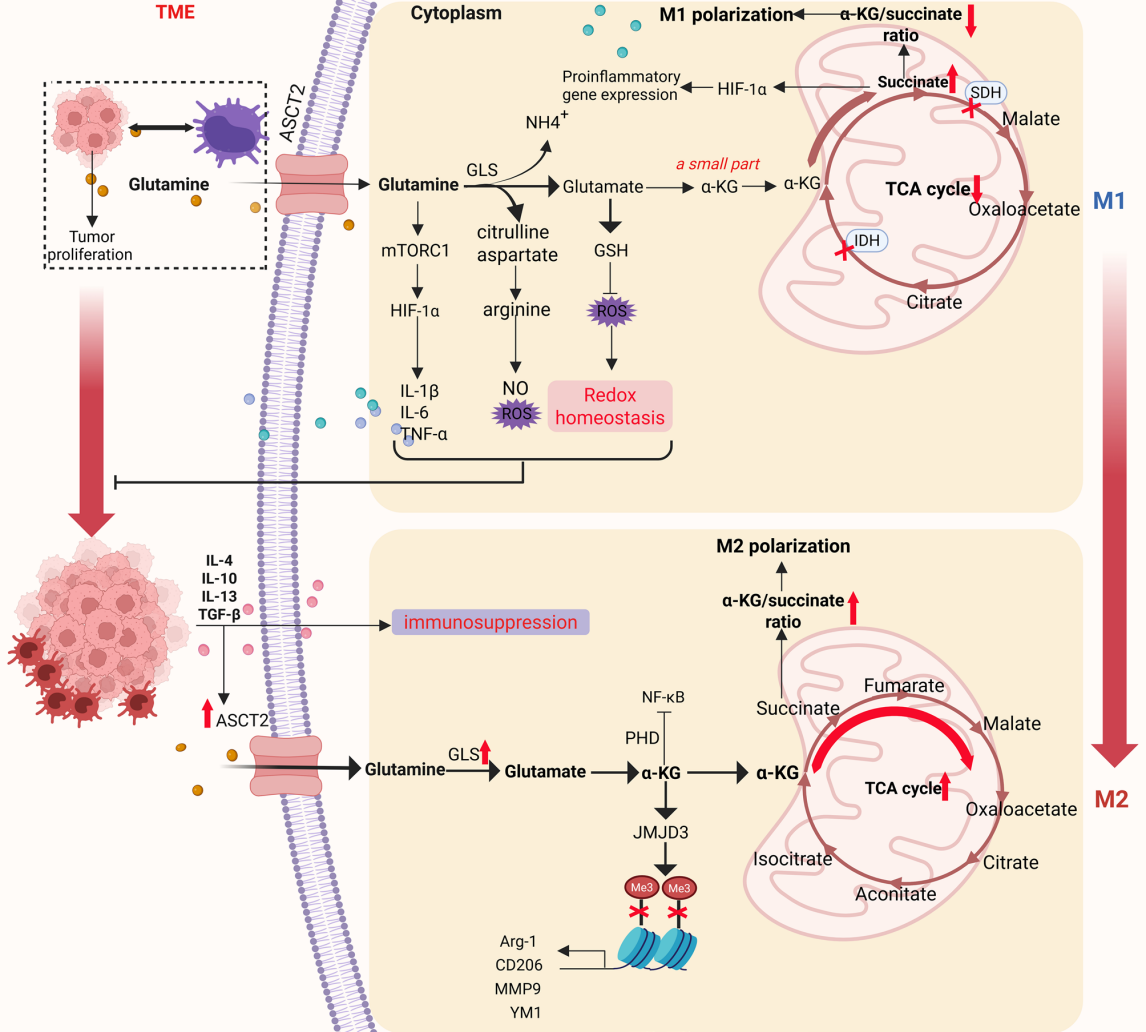
Changes of glutamine metabolism in macrophages during cancer development. Within tumor-associated inflammatory environments, macrophages utilize glutamine for the generation of glutamate, citrulline, and asparagine. A proportion of glutamate can be converted into α-ketoglutarate (α-KG), contributing to TCA cycle flux, while additional glutamate-derived metabolites, including glutathione (GSH), support redox balance and nitrogen metabolism. In certain contexts, glutamine availability has been linked to activation of signaling pathways such as mTORC1, which are associated with pro-inflammatory macrophage programs. As tumors progress and metabolic competition intensifies, macrophages may increasingly rely on glutamine metabolism as an adaptive energy and biosynthetic pathway. Enhanced α-KG production and changes in the α-KG/succinate ratio have been associated with transcriptional profiles enriched in immunoregulatory and tissue-remodeling features in subsets of tumor-associated macrophages. Importantly, these metabolic configurations appear to reflect context-dependent reprogramming rather than a fixed or irreversible transition between discrete polarization states

Nutrient scarcity in the TME and intense competition for metabolic substrates highlight the context-dependent importance of glutamine in regulating the metabolism of both tumor cells and macrophages. Moreover, glutamine and its metabolite α-KG have been reported to exert dual roles in modulating macrophage metabolic programs, reflecting the context-dependent reliance of macrophages on shifting metabolic patterns throughout tumor progression. In summary, across the spatial and temporal dynamics of tumor progression, macrophage glutamine metabolism gradually shifts from primarily supporting redox balance and immune activation toward supporting anabolic processes and context-dependent immunoregulatory functions. This metabolic reprogramming contributes to a spectrum of macrophage functional states, biasing them from tumor-inhibitory toward tumor-supportive roles in a context-dependent manner (Fig. 4).

### Other metabolic intermediates

From tumor initiation through progressive development, tumor cells and macrophages undergo dynamic metabolic reprogramming to adapt to changes in the TME. In addition to the metabolites discussed above, other intermediates, such as itaconate and tryptophan, also play indispensable roles in macrophage metabolic reprogramming.

Itaconate is a highly polar α, β-unsaturated dicarboxylic acid that does not easily penetrate the cell membrane [212]. Itaconate is predominantly produced by macrophages and other immune cells in vivo. As a tricarboxylic acid (TCA) cycle–derived metabolite, it is synthesized through the decarboxylation of cis-aconitate, which is generated from citrate via dehydration and catalyzed by aconitate decarboxylase 1 (ACOD1, also known as IRG1). Accumulating evidence indicates that itaconate is a crucial metabolite that exerts significant regulatory effects on inflammatory responses and oxidative stress [213, 214]. In the early stages of tumor development, cancer cells secrete a variety of inflammatory and growth factors into the surrounding milieu, recruiting and reprogramming diverse immune cells to shape the TME [215]. Upon stimulation by inflammatory cytokines, macrophages acquire pro-inflammatory functional features and exhibit robust expression of the immune-responsive gene 1 (IRG1, also known as ACOD1). ACOD1 catalyzes the decarboxylation of cis-aconitate to generate itaconate, thereby contributing to its accumulation within macrophages during tumor development [213, 216]. Itaconate inhibits SDH activity during the TCA cycle. The main mechanism is that itaconate has a highly similar structure to succinic acid and competitively binds to the active site of SDH. It occupies the position where succinic acid should be bound, but cannot be catalyzed by SDH itself. This prevents succinic acid from binding and oxidizing, effectively inhibiting SDH enzyme activity [217–219]. In addition, Bambouskova et al. reported that the anti-inflammatory effects of itaconate derivatives are primarily mediated through electrophilic stress and regulation of the IκBζ–ATF3 signaling axis, highlighting a mechanism beyond SDH inhibition and mitochondrial metabolic regulation [220]. Itaconate can covalently modify cysteine residues via Michael addition, thereby inhibiting KEAP1 and activating the KEAP1–NRF2 pathway to reduce oxidative stress in macrophages [212, 221]. The transport and efflux of itaconate in macrophages also involve strict regulatory mechanisms. When itaconate accumulates in macrophages with pro-inflammatory functional features, it can be transported into the cytoplasm via dicarboxylate transporters and subsequently effluxed through ABCG2, with SLC13A3 mediating its reuptake [216]. Activation of the itaconate efflux mechanism in macrophages with pro-inflammatory functional features contributes to a gradual increase in extracellular itaconate levels within the TME. Studies have shown that the concentration of itaconate in the TME increases during tumor development, and that SLC13A3 knockout significantly impairs tumorigenesis and tumor progression, indicating the necessity of itaconate for tumor proliferation. Simultaneously, itaconate can promote the alkylation of the 272 cysteine residues of PD-L1 in tumor cells, inhibiting the ubiquitination of PD-L1 and subsequent proteasome degradation, thereby increasing its stability. This enhances the binding of PD-L1 to its receptor PD-1 on T cells, thereby contributing to CD8^+^ T cell depletion and exacerbating the immunosuppressive state of the TME, ultimately promoting tumor immune escape [222]. Studies have shown that tumor cells can further enhance *Irg1* expression in TAMs via the NF-κB signaling pathway. The resulting itaconate inhibits TET DNA dioxygenase activity, suppresses the transcription of certain pro-inflammatory genes, and reduces the infiltration of CD8^+^ T cells into the TME, thereby serving a critical function in tumor immune regulation. *Irg1*^-^ deficient mice exhibited increased resistance to tumor growth, enhanced CD8^+^ T cell infiltration, and improved responses to PD-L1 blockade, indicating that *Irg1*^-^ deficient TAMs can reshape the TME [213].

Tryptophan (Trp), also referred to as β-indolylalanine, is an essential amino acid in humans. Beyond serving as a fundamental substrate for protein biosynthesis, it exerts critical functions in diverse biological processes, including cellular metabolism, inflammatory and immune regulation, and modulation of oxidative stress [223]. In the human body, tryptophan catabolism primarily proceeds through three distinct pathways: (1) the kynurenine (Kyn) pathway, which accounts for more than 95% of free tryptophan degradation; (2) the serotonin pathway, wherein a minor fraction of tryptophan is converted into neurotransmitters (e.g., 5-hydroxytryptamine, 5-HT) and neuromodulatory molecules (e.g., tryptamine); and (3) the aryl hydrocarbon receptor (AhR)-associated pathway [224–226]. In early tumor development, increased IFN-γ levels stimulate indoleamine 2,3-dioxygenase 1 (IDO1) expression in tumor cells and macrophages within the TME, thereby promoting competition for tryptophan among different cell populations. The IDO enzyme in the macrophages converts tryptophan into kynurenine. During tumor progression, tryptophan metabolism in macrophages leads to kynurenine [227–229]. Studies have shown that kynurenine acts as an endogenous ligand activating the AhR. AhR dimerizes with the aryl hydrocarbon receptor nuclear translocator (ARNT) and exerts transcriptional activity, initiating the transcription of cytokines such as IL-10 and TGF-β and biasing macrophages toward immunoregulatory and tissue-repairing functional states [230–232]. In addition, kynurenine-activated AhR can inhibit CD8^+^ T cell activation and promote the formation of an immunosuppressive microenvironment [160, 233]. Tryptophan metabolism exerts context-dependent effects on macrophage functional states, with its metabolites biasing macrophages toward immunoregulatory and tissue-repairing phenotypes that contribute to inflammation resolution and tissue homeostasis. However, under pathological conditions, such as in the TME, this shift may be co-opted to facilitate immune evasion and disease progression.

These studies highlight that itaconate and tryptophan play key regulatory roles in macrophage metabolic reprogramming, immunoregulatory functions, and tumor cell proliferation in the TME from the initial to later stages of tumor development, reflecting the multiple effects of various metabolic intermediates in macrophage metabolic reprogramming. These insights also underscore the therapeutic potential of targeting key metabolic enzymes, such as IDO1.

## Research on the strategy of targeted macrophage metabolic therapy

As the main immune cells that shape and regulate the TME, studies have shown that macrophages account for approximately 30–50% of the total number of immune cells in solid tumors [234]. Moreover, signals such as cytokines, a hypoxic environment, and metabolic substrate competition within the TME influence the metabolic reprogramming of macrophages and subsequently regulate their functions. Therefore, macrophage metabolism is an important target in tumor immunotherapy [159, 160, 235, 236]. At present, strategies targeting macrophages in cancer therapy mainly include blocking TAM recruitment, reprogramming immunosuppressive TAMs toward pro-inflammatory and tumoricidal functional states, and selectively depleting immunosuppressive macrophage subsets.

### Targeting macrophage recruitment and activation

In addition to directly targeting macrophage metabolism, several therapeutic strategies modulate TAM recruitment or activation and indirectly influence macrophage metabolic states within the TME.

CpG oligodeoxynucleotides (CpG-ODNs) are synthetic non-methylated CpG motif DNA short chains. Their primary biological mechanism involves activating TLR9 to induce a robust Th1-type immune response (IFN-α/γ, TNF-α, etc.) and stimulating macrophages to utilize glutamine and glucose as metabolic substrates. This agonist can also enhance macrophage-mediated phagocytosis by counteracting CD47-mediated “don’t eat me” signals on tumor cells, thereby promoting efficient tumor cell clearance [237].

Colony-stimulating factor-1 receptor (CSF-1 R) is a transmembrane receptor tyrosine kinase that belongs to the platelet-derived growth factor receptor (PDGFR) family and mediates downstream signaling upon binding to CSF-1. The CSF-1/CSF-1 R signaling pathway plays a crucial role in regulating the survival, proliferation, differentiation, recruitment, and functional activities of mononuclear phagocytes, including macrophages and monocytes. Continuous activation of the CSF-1/CSF-1 R pathway upregulates the macrophage transcription factor PPAR-β/δ, promotes FAO, and promotes macrophage polarization toward immunosuppressive and tissue-remodeling functional states [141, 238]. Studies have found that interference with CSF-1 R can block the CSF-1/CSF-1 R pathway of tumor cells and TAMs in the TME, alleviate the immunosuppressive microenvironment, reduce the recruitment efficiency of TAMs, and enhance the killing activity of tumor immune effector cells [239, 240]. In recent years, several inhibitors targeting the CSF-1/CSF-1 R pathway are currently under clinical evaluation (Table 2).

**Table 2:** Macrophage-targeting immunotherapies regulating TAM recruitment or activation

Therapeutic category	Target	Drug	Mechanism of action	Development stage	References
TAM recruitment blockade	CSF-1 R	PLX3397	Small-molecule CSF-1 R inhibitor reducing TAM recruitment and survival	Phase II	[241, 242]
TAM recruitment blockade	CSF-1 R	BLZ945	Selective CSF-1 R inhibitor suppressing TAM infiltration	Phase II	[243]
TAM depletion	CSF-1 R	RG7155	Anti-CSF-1 R monoclonal antibody depleting macrophages	Phase I	[244]
Innate immune activation	TLR9	CpG-ODN	TLR9 agonist activating innate immune responses	Phase I	[245]
Innate immune activation	TLR3	Poly ICLC	Synthetic dsRNA agonist stimulating immune activation	Phase I/II	[246]
Immunometabolic checkpoint	IDO1	Epacadostat	IDO1 inhibitor targeting tryptophan metabolism and immune suppression	Phase II	[247]
Macrophage activation	CD40	APX005M	CD40 agonistic antibody activating APCs	Phase II	[248]
Macrophage activation	CD40	CDX-1140	CD40 agonist enhancing macrophage and dendritic cell activation	Phase II	[249]
Phagocytosis checkpoint	CD47	ALX148	CD47–SIRPα blockade enhancing macrophage-mediated phagocytosis	Phase II	[250]

### Targeting fatty acid oxidation–associated metabolic reprogramming

Fatty acid oxidation (FAO) is an important metabolic pathway that contributes to macrophage functional polarization within the TME.

CD40 (TNFRSF5) is a type I transmembrane glycoprotein primarily expressed on antigen-presenting cells (APCs), including monocytes, macrophages, and dendritic cell subsets, where it transduces essential immunoregulatory signals through interaction with its ligand CD40L. Upon CD40L binding at the cell surface, CD40 recruits tumor necrosis factor receptor-associated factors (TRAFs), thereby activating downstream NF-κB and PI3K signaling cascades and mediating immunoregulatory signaling [251].

Prior research has indicated that the CD40 agonist FGK45 can simultaneously activate FAO and glutamine metabolism in macrophages. FAO-derived acetyl-CoA promotes histone acetylation at the promoters and enhancers of pro-inflammatory genes, thereby driving the anti-tumor activity of macrophages. In addition, CD40 signaling enhances glutamine metabolism to support lactate production, and lactate, in turn, sustains FAO homeostasis by fine-tuning the intracellular NAD^+^ /NADH ratio. Notably, when FAO is inhibited in macrophages, the FGK45-mediated induction of anti-tumor and pro-inflammatory genes in TAMs is markedly attenuated, while its suppressive effect on pro-tumor genes is also markedly diminished [252]. Several CD40 agonists are currently under clinical evaluation (Table 2).

### Targeting mitochondrial respiration and oxidative phosphorylation

Mitochondrial oxidative phosphorylation (OXPHOS) plays a key role in macrophage metabolic programming and functional regulation within the TME.

Clodronate-containing liposomes formulated with DOTAP (Clo-Lipo-DOTAP) are relatively mature macrophage scavengers that can be phagocytosed by TAMs in the body, thereby leading to the release of clodronate and its conversion into non-hydrolyzable ATP analogs, which block the mitochondrial electron transport chain and inhibit the OXPHOS pathway of TAMs, thereby leading to selective depletion of immunosuppressive TAMs within the TME. Goulielmaki et al. employed Clo-Lipo-DOTAP to treat solid tumors in mice, demonstrating its ability to deplete immunosuppressive TAM subsets within tumor tissues and markedly enhance mouse survival [253].

Metformin is a clinically used hypoglycemic drug. Studies in preclinical models have shown that metformin can reprogram immunosuppressive macrophages toward pro-inflammatory functional states, partly through activation of AMP-activated protein kinase (AMPK) and regulation of the NF-κB signaling pathway. In animal models, metformin treatment has been associated with reduced numbers of bone marrow-derived suppressor cells (MDSCs) and regulatory T cells (Tregs), contributing to inhibition of tumor growth and metastasis [254]. Notably, although metformin affects multiple immune compartments, including MDSCs and Tregs, preclinical studies indicate effects on TAMs. However, most studies do not include macrophage-specific perturbation or depletion controls, so direct TAM-intrinsic effects remain to be fully validated. Reported doses and models vary across studies, highlighting the need for more detailed investigation of TAM-specific mechanisms in vivo. These findings suggest that metformin may modulate macrophage metabolism in the tumor microenvironment, although its therapeutic potential in humans requires clinical validation.

IACS-010759, a clinical-stage complex I inhibitor of the mitochondrial electron transport chain, impedes tumor proliferation and migration by inhibiting OXPHOS in both tumor and immune cells [255, 256].

### Targeting glutamine metabolism

Glutamine metabolism is another important metabolic pathway that supports tumor growth and contributes to the immunosuppressive phenotype of TAMs.

6-Diazo-5-oxo-L-norleucine (L-DON) is a first-generation anti-glutamine metabolism agent that suppresses the activity of various glutamine-dependent enzymes, thereby interfering with glutamine metabolism in tumors and immune cells [257, 258]. Owing to the strong toxicity of L-DON, researchers have developed its precursor JHU-083. After entering the TME, JHU-083 is activated into L-DON and targets glutamine metabolism to inhibit tumor growth and immunosuppressive TAM reprogramming in prostate and bladder tumors, thereby promoting an anti-tumor immune response [259]. In contrast, L-DON can inhibit the release of tumor-derived cytokines (such as granulocyte CSF-3), thereby reducing the production of regulatory immune cells, promoting the expansion and activation of pro-inflammatory macrophage populations, and enhancing antigen presentation to CD8^+^ T cells [260]. These findings suggest that targeting glutamine metabolism in tumor-associated macrophages could be a promising strategy in clinical cancer immunotherapy.

### Targeting arginine metabolism

Arginine metabolism also contributes to the immunosuppressive properties of TAMs within the TME.

Based on Phase I clinical data, INCB001158, an Arginase inhibitor, has been shown to increase arginine levels in the TME, thereby alleviating immune suppression and restoring the function of immune effector cells. In combination with gemcitabine, it has demonstrated significant therapeutic benefits in patients with cholangiocarcinoma [261].

Although numerous investigational agents have shown promise in treating cancer by targeting macrophage metabolism, the clinical translation of these strategies remains limited due to tumor heterogeneity, disease complexity, and microenvironmental variability. Related targets for enhancing cancer treatment through the regulation of macrophage metabolism need to be further explored and developed (Table 3).

**Table 3:** Therapeutic agents targeting macrophage metabolic pathways in tumors

Metabolic pathway	Target	Drug	Mechanism of action	Development stage	References
mTOR signaling and metabolic reprogramming	mTOR	Rapamycin	Inhibits mTOR signaling and alters macrophage metabolic programming	Phase II	[262]
Energy sensing and mitochondrial respiration	AMPK/Complex I	Metformin	Activates AMPK and suppresses mitochondrial respiration, promoting pro-inflammatory macrophage polarization	Phase II	[263, 264]
	ETC Complex I	IACS-010759	Inhibits mitochondrial complex I, suppressing oxidative phosphorylation	Phase I	[255]
Pyruvate metabolism and mitochondrial substrate utilization	Pyruvate dehydrogenase kinase (PDK)	Dichloroacetate	Inhibits PDK and shifts metabolism toward mitochondrial oxidation	Phase II	[265, 266]
	Mitochondrial pyruvate carrier (MPC)	UK5099	Blocks pyruvate transport into mitochondria and alters metabolic flux	Preclinical	[267]
Lactate transport and metabolic communication	Monocarboxylate transporter 1 (MCT1)	AZD3965	Inhibits lactate transport and disrupts metabolic crosstalk in the TME	Phase I	[268]
Glutamine metabolism	Glutamine-dependent enzymes	JHU-083	DON prodrug that suppresses glutamine-dependent metabolic pathways	Preclinical	[260]
	Glutaminase (GLS)	CB-839	Inhibits glutaminase and suppresses glutamine metabolism	Phase II	[269]
Arginine metabolism	Arginase	INCB001158	Inhibits arginase and restores arginine availability in the TME	Phase I	[261]

## Discussion and perspective

### Controversies and limitations

Despite the conceptual utility of classical polarization models, the binary M1/M2 framework increasingly fails to capture the dynamic and context-dependent nature of macrophage states within the tumor microenvironment. Accumulating evidence from single-cell RNA sequencing, spatial transcriptomics, and metabolomic profiling demonstrates that tumor-associated macrophages exist along a multidimensional continuum rather than discrete polarization states [270]. Their phenotypes are shaped by combinatorial microenvironmental cues, metabolic fluxes, and temporal progression, leading to overlapping and hybrid functional programs that defy strict classification.

Similarly, dichotomous interpretations such as “glycolytic versus FAO-dependent metabolism,” “early-stage versus late-stage tumor context,” or “nutrient-abundant versus nutrient-depleted conditions” may oversimplify the intricate metabolic plasticity observed in vivo. Macrophage metabolic programs are not mutually exclusive but often coexist or dynamically shift in response to evolving environmental pressures.

Furthermore, although emerging single-cell and spatial technologies have greatly advanced our understanding, the integration of metabolic flux data with transcriptomic and functional readouts remains technically challenging. Current approaches may not fully resolve transient metabolic states or causal relationships between metabolic intermediates and immune function. Therefore, a more nuanced framework that integrates multidimensional data and recognizes macrophage heterogeneity as a spectrum rather than a binary classification is required.

### Conclusion and perspective

In conclusion, this review provides an integrated and updated perspective on how the dynamic temporal progression and spatial heterogeneity of the tumor microenvironment orchestrate macrophage activation, polarization, and metabolic reprogramming throughout tumor development. By carefully dissecting the reciprocal relationships between microenvironmental cues and macrophage metabolic states, we underscore that macrophage metabolism functions as a central regulatory hub linking environmental stress, immune plasticity, and tumor immune evasion.

Importantly, we identify several key metabolic intermediates that not only serve as indicators of microenvironmental alteration but also act as potent modulators capable of redirecting macrophage functional phenotypes. The recognition of these metabolites as actionable nodes reveals new layers of complexity in tumor immunoregulation and provides mechanistic insights into how metabolic reprogramming shapes antitumor or protumor macrophage behaviors.

Furthermore, by summarizing emerging therapeutic strategies and agents targeting macrophage metabolism, this review highlights the growing feasibility of manipulating metabolic circuits to enhance the efficacy of cancer immunotherapies. Targeting macrophage metabolism represents a promising avenue to overcome resistance mechanisms, improve responsiveness to existing immunotherapies, and expand therapeutic options for tumors traditionally considered immunologically “cold.”

Collectively, this work not only synthesizes current knowledge but also defines conceptual frameworks and research priorities for future studies. It emphasizes the critical importance of macrophage-centered metabolic intervention as a transformative strategy with the potential to refine tumor immunotherapy and ultimately improve clinical outcomes.

Nevertheless, several limitations warrant consideration. First, the metabolic phenotypes of tumor-associated macrophages are highly dynamic and context-dependent, and the simplified dichotomous classification of macrophage polarization may not fully capture their functional complexity in vivo [34, 271]. Second, although multiple metabolic intermediates have been proposed as regulatory nodes, their causal roles and spatiotemporal dynamics within heterogeneous tumor microenvironments remain insufficiently defined [63]. In addition, most mechanistic insights are derived from preclinical models, and the translational robustness of metabolism-targeted strategies requires further validation in well-designed clinical studies. Technical variability in metabolic profiling and the lack of standardized approaches for in situ metabolite quantification also present challenges that may affect reproducibility and comparability across studies.

In light of these limitations, integrating emerging technologies with drug development will be a key strategy for advancing cancer immunotherapy. Continuous advancements in mass spectrometry have provided researchers with powerful tools for elucidating the mechanisms linking TME metabolites and macrophage metabolic reprogramming. Meanwhile, the development of microenvironment-responsive nanomedicines, gene-editing tools (e.g., CRISPR-Cas9 targeting metabolic enzymes), and combination therapies with other monoclonal antibodies holds promise for precise tumor modulation. Integrating multiomics analyses with organoid models will further accelerate the clinical translation of metabolism-targeted cancer therapies. Although emerging CAR-T and TCR-T technologies are developing rapidly, their therapeutic effects on solid tumors are limited, and they exhibit poor tumor infiltration and significant toxic side effects clinically [272, 273]. Macrophages, the main immune cell population in the TME, rely on a continuous supply from the myeloid system [49, 240]. Therefore, CAR-macrophage technology will also be a potential means of tumor immunotherapy. Building on these emerging technologies, we anticipate that future studies will more deeply investigate the role of macrophage metabolism in tumor immunity while facilitating the clinical translation of related therapeutics.

## Data Availability

Not applicable.
